# TRIM36 inhibits tumorigenesis through the Wnt/β-catenin pathway and promotes caspase-dependent apoptosis in hepatocellular carcinoma

**DOI:** 10.1186/s12935-022-02692-x

**Published:** 2022-09-06

**Authors:** Qing Tong, Mingyu Yi, Panpan Kong, Lin Xu, Wukui Huang, Yue Niu, Xiaojing Gan, Huan Zhan, Rui Tian, Dong Yan

**Affiliations:** 1 Department of Hepatopancreatobiliary Surgery, Affiliated Cancer Hospital of Xinjiang Medical University, Urumqi, 830011 Xinjiang China; 2 The Third Affiliated, Teaching Hospital of Xinjiang Medical University, Urumqi, Xinjiang China; 3 The Third Xiangya Hospital, Central South University, Changsha, Hunan China; 4 National Cancer Center/National Clinical Research Center for Cancer/Cancer Hospital & Shenzhen Hospital, Chinese Academy of Medical Sciences and Peking Union Medical College, Shenzhen, China; 5 Department of Biliary-Pancreatic Surgery, Affiliated Tongji Hospital, Tongji Medical College, Huazhong University of Science and Technology, Wuhan, Hubei China

**Keywords:** TRIM36, Caspase-3, Caspase-7, Apoptosis, RNA-seq, Wnt/β-catenin pathway

## Abstract

**Background:**

Hepatocellular carcinoma (HCC) is the most common type of primary liver cancer and has an extremely poor prognosis. We aimed to determine the latent relationships between TRIM36 regulation of apoptosis and the Wnt/β-catenin pathway in HCC.

**Methods:**

Immunohistochemistry and western blotting were used to characterize the aberrant expression of TRIM36 in HCC and adjacent tissues. Clinical information was analyzed using Kaplan–Meier and Cox methods. RNA-seq of potential targets was conducted to detect the regulation of TRIM36. Apoptosis assays and cellular proliferation, invasion and migration were conducted in a loss- and gain-of-function manner in cultured cells to determine the biological functions of TRIM36*.* A rescue experiment was conducted to confirm the role of Wnt/β-catenin signaling in TRIM36 regulation. Finally, in vivo experiments were conducted using cell line-derived xenografts in nude mice to validate the central role of TRIM36 in HCC.

**Results:**

TRIM36 expression was significantly downregulated in HCC tissues compared to adjacent non-tumor tissues. TRIM36 repressed the proliferation, migration, and invasion of Huh7 and HCCLM3 cells, whereas it stimulated apoptosis. Wnt/β-catenin signaling was inhibited by TRIM36, and rescue experiments highlighted its importance in HCC proliferation, migration, and invasion. In vivo experiments further confirmed the effects of sh-TRIM36 on HCC tumorigenesis, inhibition of apoptosis, and promotion of Wnt/β-catenin signaling.

**Conclusion:**

Our study is the first to indicate that TRIM36 acts as a tumor suppressor in HCC. TRIM36 activates apoptosis and inhibits cellular proliferation, invasion, and migration via the Wnt/β-catenin pathway, which may serve as an important biomarker and promising therapeutic target for HCC.

**Supplementary Information:**

The online version contains supplementary material available at 10.1186/s12935-022-02692-x.

## Background

Hepatocellular carcinoma (HCC) is the most common type of primary hepatic malignancy. It accounts for approximately 850,000 deaths annually worldwide [1, 2]. Despite significant progress in research and treatment, because patients exhibit no obvious symptoms, HCC still has an extremely unfavorable prognosis, characterized by diagnosis at advanced stages, malignant growth, and early metastasis [3–5]. Recently, with an understanding of the mechanisms and risk factors (such as hepatitis B virus) underlying cirrhosis, multiple genomic and epigenomic alterations have been identified in HCC [4, 6, 7]. Rebouissou et al. distinguished driver genes in HCC, including those involved in telomere maintenance, P53/cell-cycle regulation, AKT/mTOR, MAP kinase, epigenetic factors, oxidative stress, and the Wnt/β-catenin pathway [3]. However, the complex connections and multistep processes involved in HCC are still important conundrums needed to dissect, providing novel biomarkers and therapeutic targets.

Wnt/β-catenin signaling plays a pivotal role in hepatic development and homeostasis, including hepatoblast differentiation and liver zonation; correspondingly, it contributes to the pathogenesis of liver malignancies [8, 9]. The cytoplasmic β-catenin pool plays an effector role in Wnt signaling [8, 10]. Multiple studies have revealed a correlation between β-catenin activation and immune escape in HCC [11, 12]. HCC is characterized by an 11–37% incidence of activating mutations in *CTNNB1*, the gene coding for β-catenin, leading to its stabilization and nuclear translocation [13, 14]. In contrast, the inactivation of Wnt ligand inhibitors doubles the activation of the Wnt/β-catenin pathway in HCC [3]. The activation of Wnt/β-catenin signaling increases liver carcinogenesis sensitivity, stemness, chemoresistance, tumor recurrence, and metastasis [15].

Tripartite motif family (TRIM) proteins share a canonical RING finger, B-box, and coiled-coil (RBCC) structure [16, 17]. The RING finger, an E3 ubiquitin ligase, often regulates ubiquitination [18, 19]. One or two specific C-terminal domains following the classic RBCC structure are unique to members of the TRIM family [20, 21]. This family consists of over 80 members that play roles in multiple diseases, including viral infection, neurological diseases, and cancers [22]. It has been shown that TRIM36 mediates multiple protein–protein interactions in DNA repair and signal transduction, leading to the regulation of cellular proliferation, immunity, and apoptosis [23, 24].

TRIM36 (also called HAPRIN) is expressed at different levels in diverse tissues [25]. The *TRIM36* gene is located on chromosome 5q22.3, a tumor suppressor region [26, 27]. Of note, immunohistochemistry has shown that TRIM36 has a filamentous cytoplasmic organization. And it has been associated with microtubule-binding [28]. Maternal knockdown of TRIM36 leads to failed microtubule assembly in ventralized embryos and *Xenopus* cortical rotation [29]. Similarly, the triggering of Wnt/β-catenin signaling during early embryonic development is dependent on microtubule-dependent cytoplasmic localization. Notably, the microtubule-associated protein regulator of cytokinesis 1 (PRC1) is associated with early HCC recurrence and is a direct target of Wnt signaling [30]. TRIM36 was found to exert a pro-apoptotic function in prostate cancer (PC) [27], while anti-Wnt antibodies were demonstrated to decrease apoptosis in multiple cancers, including HCC [31, 32]. Based on these data, we further study latent relationships between TRIM36 regulation of apoptosis and the Wnt/β-catenin pathway for the first time, and provide a novel biomarker and therapeutic target in HCC.

## Materials and methods

### Subjects

Patients admitted to the Affiliated Cancer Hospital of Xinjiang Medical University between September 2014 and March 2021 who met the following criteria were enrolled: (1) complete medical records, (2) post-operative histological diagnosis of HCC, and (3) hepatectomy. The exclusion criteria were: (1) presence of other malignancies or AIDS and (2) fragmented clinical or pathological data. Ultimately, 92 patients with HCC were enrolled. All provided informed consent before inclusion. We collected clinical information: age, sex, HBV infection status, cirrhosis history, AFP level before surgery, Edmondson grade, and TNM tumor stage. We also collected imaging data: tumor size (cm, length × width), vascular invasion status, and tumor numbers. Patient follow-up data were recorded: recurrence-free survival (RFS) (months after surgery), survival status, and overall survival (OS) (months after surgery). Recurrence was primarily assessed by imaging. RFS was defined as the time between surgery and the date of recurrence or failure of follow-up. OS was defined as the time between surgery and death due to HCC or date without follow-up. All experiments followed the ethical principles stipulated in the 1964 Declaration of Helsinki and its subsequent revisions. The study was approved by the Institutional Research Ethics Committee of the Affiliated Tumor Hospital of Xinjiang Medical University(K-2021024). Patient characteristics are provided in Table 1.

**Table 1:** Correlation analysis between the clinical features and TRIM36 expression in HCC

Characteristics of patients		NO.of patients	TRIM36 level		*p* value
			Low	High	
Age (y)					0.357
< 60		70	40	30	
≥ 60		22	15	7	
Gender					0.686
Male		57	35	22	
Female		35	20	15	
HBV infection					0.257
Absent		15	7	8	
Present		77	48	29	
Cirrhosis					0.637
Absent		35	22	13	
Present		57	33	24	
AFP level (ng/mL)					0.159
< 20		25	12	13	
≥ 20		67	43	24	
Tumor size (cm)					** < 0.001**
< 5		37	12	25	
≥ 5		55	43	12	
Vascular invasion					** < 0.001**
Absent		53	17	36	
Present		39	38	1	
Edmondson-Steiner grading					0.560
I+ II		64	37	27	
III + IV		28	18	10	
TNM tumor stage					** < 0.001**
I + II		47	11	36	
III + IV		45	44	1	

*HBV* hepatitis B virus, *AFP* alpha fetoprotein, *TNM* tumor-node-metastasis, Significant values are in bold

### Immunohistochemistry (IHC)

Sections from paraffin blocks were first dewaxed and then boiled with citrate solution, incubated with H_2_O_2_ for 10 min, and primary antibodies against TRIM36 (1:200, Sigma, USA), cleaved caspase-3 (1:20, Sigma), Ki-67 (1:5000, Proteintech, China), and β-catenin (1:2000, Proteintech). After washing, secondary antibodies were added. After washing, sections were stained with diaminobenzidine solution and hematoxylin. Sections were washed, dehydrated, and sealed with neutral balsam. Images were taken using an AXIO Vert A1 (Carl Zeiss AG, Germany) and ZEN software at magnifications of 100× and 200× .

Immunostaining intensity was scored as 0 to 3 (0, no staining; 1, weak staining; 2, moderate staining; and 3, intense staining). Stained area scores ranged from 0 to 4 (0, no staining; 1, 1–25%; 2, 26–50%; 3, 51–75%; and 4, more than 75% of tumor cells stained). The final IHC score was calculated as staining intensity × area score. Each slide was evaluated by two independent pathologists who were blinded to the sample origin.

### RNA isolation and RT-qPCR

TRIzol reagent (Thermo Fisher Scientific, Waltham, MA, USA) was added to tissues or cells. Chloroform was then added to the samples, followed by incubation for 5 min at room temperature. Then, centrifugation at 12,000 *g* × 15 min at 4 °C was performed followed by harvest of the aqueous phase. Isopropanol was added next and centrifuged at 12,000 *g* for 10 min at 4 °C. The precipitate was harvested, washed with 75% ethanol, air dried, and resuspended in RNase-free water. Complementary DNA was synthesized using the Evo M-MLV RT Kit with gDNA Clean for qPCR II, according to the manufacturer’s instructions (Accurate Biology, China). The cDNA was amplified using the SYBR^®^ Green Premix Pro Taq HS qPCR Kit (Accurate Biology, China), according to the instruction, using pre-denaturation at 95 ℃ for 30 s; amplification at 95 ℃ for 5 s and 60 ℃ for 34 s, 40 cycles; melting at 95 ℃ for 15 s, 60 ℃ for 60 s, and 95 ℃ for 15 s, 1 cycle. *ACTB* was used as an internal reference gene. Data were collected and analyzed using a Lightcycler96 instrument and the 2^–∆∆Ct^ method was used for analysis. Primers are listed in Additional file 1: Table S1.

### Protein extraction

For total protein, lysis solution with radioimmunoprecipitation assay (RIPA, Beyotime) lysis and 1% phenylmethanesulfonyl fluoride (PMSF, Beyotime) were added to the tissues or cells on ice, and 30 min later, the lysate was centrifuged at 15,000 rpm for 15 min, and the supernatant was harvested. A nuclear protein, nuclear protein, and cytoplasmic protein extraction kit (Beyotime, China) was used to isolate nuclear proteins according to the manufacturer’s instructions. BCA kits (Beyotime, China) were used to measure protein concentrations. Protein samples were boiled with 25% v/V loading buffer (Beyotime, China) at 100 °C for 5 min and stored at − 20 °C.

### Western blotting

Proteins were separated by SDS-PAGE. After electrophoresis, gels were cut and transferred to PVDF membranes. Membranes were blocked with 5% w/V evaporated milk in PBST. Diluted primary antibodies were added and incubated overnight at 4 °C. Primary antibodies were against TRIM36 (1:1000, Sigma), BAX (1:10,000, Proteintech), and BCL2 (1:2000, Proteintech), caspase-3 (1:1000, Abclone), cleaved caspase-3 (1:200, Sigma), caspase-7 (1:5000, Proteintech), PARP1 (1:1000, Abclone), MMP-9 (1:500, Proteintech), cyclin D1 (1:1500, Proteintech), β-catenin (1:20,000, Proteintech), active β-catenin (1:1000, Cell signaling technology), c-JUN (1:2000, proteintech), Histone H3 (1:10,000, Proteintech), Actin (1:10,000, Proteintech). Thereafter, membranes were incubated with horseradish peroxidase-conjugated goat anti-rabbit IgG (H + L) or HRP-goat anti-mouse IgG (H + L) secondary antibodies (Proteintech) for 50 min at room temperature. Chemiluminescence was measured using Super Signal West Atto (Thermo Fisher Scientific). Blots were imaged with Amersham Imager 600 software and analyzed using ImageJ software.

### Cell culture and transfection

Human normal liver cell LO2, human hepatoma cell lines PLC/PRF/5, Huh7, Hep-G2, HCCLM3, and MHCC-97H were purchased from the Chinese Academy of Sciences. All were cultured in DMEM supplemented with 10% fetal bovine serum (FBS), penicillin (100 U/ml), and streptomycin (100 mg/ml) (all from Gibco) at 37 °C and 5% CO_2_ in a cell incubator. The siRNA targeting TRIM36 and β-catenin, and the corresponding si-control were purchased from RIB-BIO (China) and transfected into Huh7 and HCCLM3 cell lines using Lipofectamine 3000 (Thermo Fisher Scientific), according to the manufacturer’s instructions. Transfection efficiency was determined using western blotting and qRT–PCR. The sequences are listed in Additional file 1: Table S1. Purified Wnt ligand WNT3a (98.68%) (Cat: HY-P70453) were purchased from MedChemExpress (NJ, USA), and treated cells at 100 ng/mL concentration.

### Lentiviral transduction

To establish stable cell lines, lentiviral vectors for increasing TRIM36 expression levels and a vector-only control were purchased from GENECHEM (China). Lentiviral particles were transduced into HCCLM3 and Huh7 cells, followed by puromycin screening for 2 weeks. Elevated expression of TRIM36 was identified by western blotting and qRT–PCR; only cells with stably elevated TRIM36 expression were used for further examination.

### Cell counting

Cells expressing the target gene were seeded in 96-well plates with 100 μL complete medium. Thereafter, 10 μL of CCK-8 reagent (Dojindo Laboratories, Japan) was added to each well at different time points (0, 1, 2, 3, 4, 5, and 6 d), according to the manufacturer’s instructions. After incubation at 37 °C for 1 h, optical density was measured at 450 nm.

### Colony formation

HCCLM3 and Huh7 cells with desired expression patterns were harvested and resuspended. Cells were seeded at 1000/well into six-well plates for colony formation. After approximately 2 weeks of culture, cells were fixed with 4% paraformaldehyde for 30 min and stained with 0.1% crystal violet for 20 min at room temperature in the dark. Visible colonies (diameter > 0.1 mm were photographed and counted.

### Migration and invasion assays

For the invasion assay, Matrigel film (Corning, USA) was prepared before cell inoculation; a similar procedure was employed for the migration and invasion assays. Cells with targeted gene expression were collected and resuspended in serum-free medium to the same density. Cell suspensions (200 μL) were seeded in the upper chamber and 750 μL of medium with 10% FBS was added to the lower chamber. Cells were cultured for 48 h, fixed with formaldehyde, and stained with 0.1% crystal violet. Cells in the upper chamber were removed, and cells that crossed the membrane were retained. Images were taken with an EVOS XL Core Instrument (AMEX1000, Thermo Fisher Scientific, USA) at 100× and 200× magnifications. Cells were counted using ImageJ software for five fields at 200× magnification per chamber (n = 1) in triplicate. Images are shown at 100× magnification.

### TUNEL apoptosis assay

Cells expressing TRIM36 were seeded into 96-well plates. Cells were fixed with 4% paraformaldehyde and incubated with 0.1% Triton X-100 for 2 min. After the addition of 50 μL TUNEL solution per well, cells were incubated in the dark at 37 °C for 60 min and washed twice with PBS. Fluorescence was captured using an AXIO Vert A1 fluorescence microscope (Carl Zeiss AG, Germany) and ZEN software at 200× magnification.

### Annexin V/PI apoptosis assay

Apoptosis was measured by annexin V/PI staining (Thermo Fisher Scientific, USA). Cells with altered TRIM36 expression were harvested and resuspended in 200 μL of buffer containing annexin V and propidium iodide (PI) for 15 min at 37 °C in the dark. Staining intensities were measured by flow cytometry to distinguish between healthy, apoptotic, and dead cells. Data were analyzed using FlowJo version 10.8.0.

### RNA-Seq

TRIM36 was knocked down in Huh7 cells using siRNA (RIB-BIO, China) in parallel with control cells with the control siRNA. TRIzol™ reagent (Thermo Fisher Scientific, USA) was used to purify total RNA from cells. An Illumina 6000-PE150 (Illumina, USA) was used for RNA sequencing. Data were annotated to the human genome and differentially expressed mRNAs were analyzed using R software.

### Bioinformatics

Heatmap and volcano plots were drawn using R based on differential gene analysis. Gene Oncology (GO) was used to perform functional analyses of differentially expressed genes. The Kyoto Encyclopedia of Genes and Genomes (KEGG) was used to identify enriched pathways. Gene set enrichment analysis (GSEA) was performed to identify significantly enriched genes using GSEA software (Broad Institute, Cambridge, MA).

### Murine xenograft model

Five-week-old BALB/c male nude mice were purchased from the Chinese Academy of Sciences. Huh7 cells were transduced with either lenti-sh-TRIM36 (GENECHEM) or lenti-sh-NC (GENECHEM). Cells were suspended in serum-free DMEM and mixed with Matrigel (Corning) in a 1:1 ratio. Cells with lenti-sh-TRIM36 were injected subcutaneously into the right dorsum, and cells containing lenti-sh-NC were injected into the left dorsum as controls. Tumor width and length were measured every 3 days, and volumes were calculated according to the formula V = length × width^2^ × 0.52. Mice were euthanized with 5% Pentobarbital and necropsied after 3 weeks. Weights of mice and their subcutaneous masses were recorded. Excised tissues were stored separately for different measurements.

### Statistical analysis

For HCC patients, clinical information was recorded and analyzed. A TRIM36 IHC score of 4 + was set as the cutoff, as the mean score in HCC tumor samples was 3.66. Thus, a score ≤ 4 represents a low expression level of TRIM36 and could be used to divide patients into comparable groups. Differences between groups were analyzed using the Kaplan–Meier method with the log-rank test. Cox univariate analysis was conducted for TRIM36 expression and other indicators commonly used for clinical prognosis. Significant variables (p < 0.05) were selected for Cox multivariate analysis, with a statistically significant p-value of < 0.05. At least three replicates were performed for both in vitro and in vivo assays. Data are presented as means ± standard deviations. Student’s *t*-test was used for comparisons between two groups. ANOVA was used to measure the differences between groups when they numbered three or more. All analyses were performed using SPSS software (Munich, Germany). Figures were generated using GraphPad Prism 8 (San Diego, CA, USA). Software used is shown in Additional file 2: Table S2.

## Results

### TRIM36 expression is downregulated in HCC and correlates with poor clinical outcomes

**Fig. 1:**
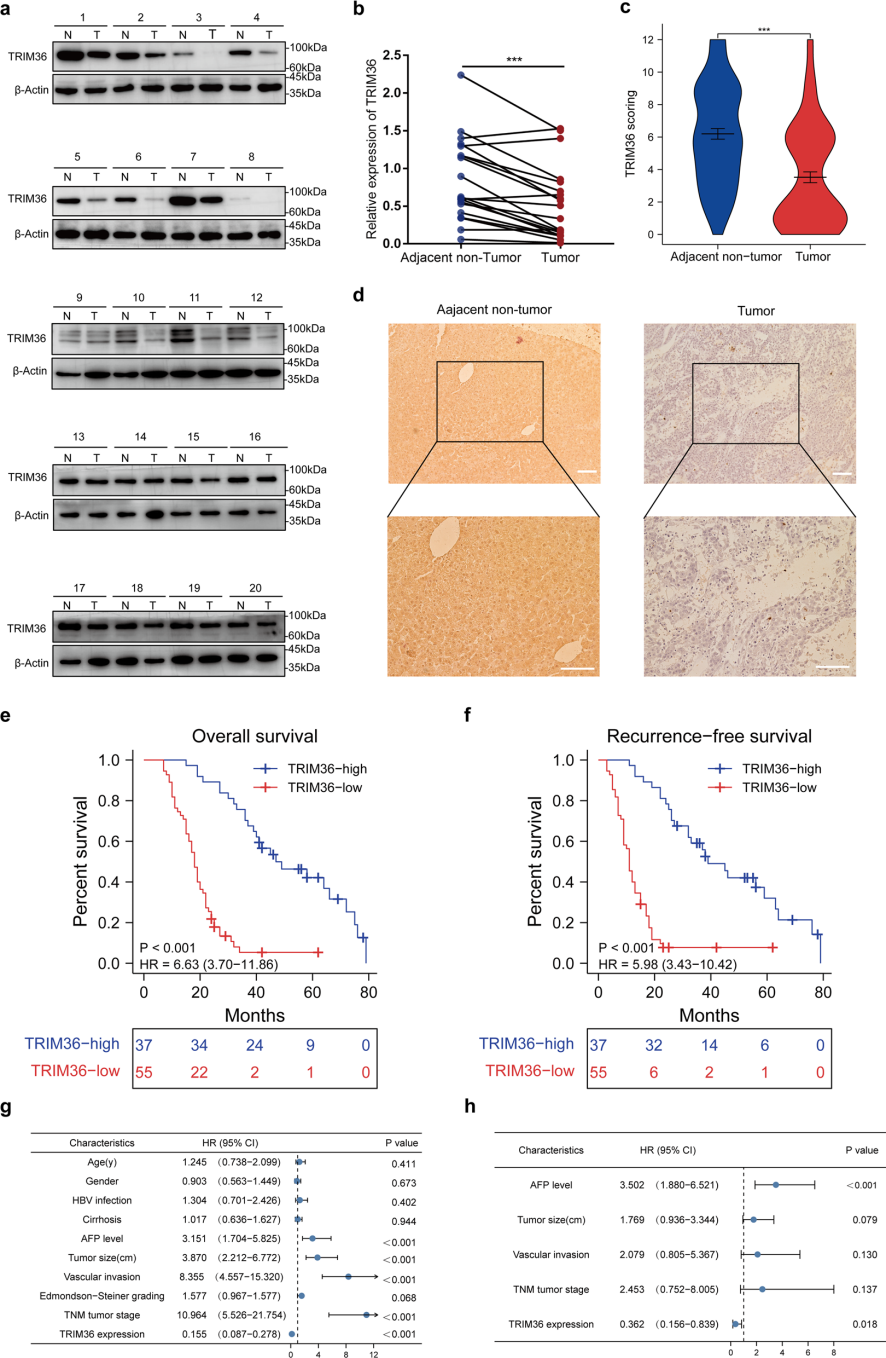
TRIM36 expression is downregulated in HCC and correlates with poor clinical outcomes. **a** Western blotting analysis of TRIM36 expression in HCC and adjacent non-tumor tissues. N = adjacent non-tumor tissues, T = HCC tissues. **b** Graph showing TRIM36 expression in HCC and adjacent non-tumor tissues, p value was calculated using two-tailed paired Student’s *t*-test, n = 20 independent repeats. **c** Violin plot graph showing the immunohistochemistry staining scores of TRIM36 in HCC and adjacent non-tumor tissues. *P* value was calculated using two-tailed paired Student’s *t*-test**,** n = 92 independent repeats. **d** Immunohistochemistry image showing the expression of TRIM36 in HCC and adjacent non-tumor tissues. Scale bars, 100 μm. **e** Kaplan–Meier analysis of TRIM36 (N_TRIM36-high_ = N_TRIM36>4_ = 37, N_TRIM36-low_ = N_TRIM36≤4_ = 55) for overall survival (OS). **f** Kaplan–Meier analysis of TRIM36 (N_TRIM36-high_ = N_TRIM36>4_ = 37, N_TRIM36-low_ = N_TRIM36≤4_ = 55) for recurrence-free survival (RFS). **g-h** Univariate and multivariate Cox analysis of OS. ***P < 0.001

Western blotting showed that TRIM36 was significantly downregulated in HCC tissues compared with para-cancerous control tissues (Fig. 1a, b). Ninety-two HCC patients (57 men and 35 women) were enrolled in this study, and the expression levels of TRIM36 in HCC tissues and adjacent non-tumor tissues were detected by IHC, showed a significant downregulation in HCC tissues (Fig. 1c, d). To assess the prognostic potential of TRIM36 expression, we performed Kaplan–Meier analyses of post-operative patients with sufficient clinical follow-up. The downregulation of TRIM36 correlated with worse OS and RFS (Fig. 1e, f). Based on Cox variable analysis, TRIM36 expression was an independent prognostic factor for both OS and RFS (Fig. 1g, h and Additional file 3: Fig. S1a, b). Taken together, these results suggest a possible tumor suppressor role for TRIM36 in HCC.

### TRIM36 represses HCC proliferation, migration, and invasion

**Fig. 2:**
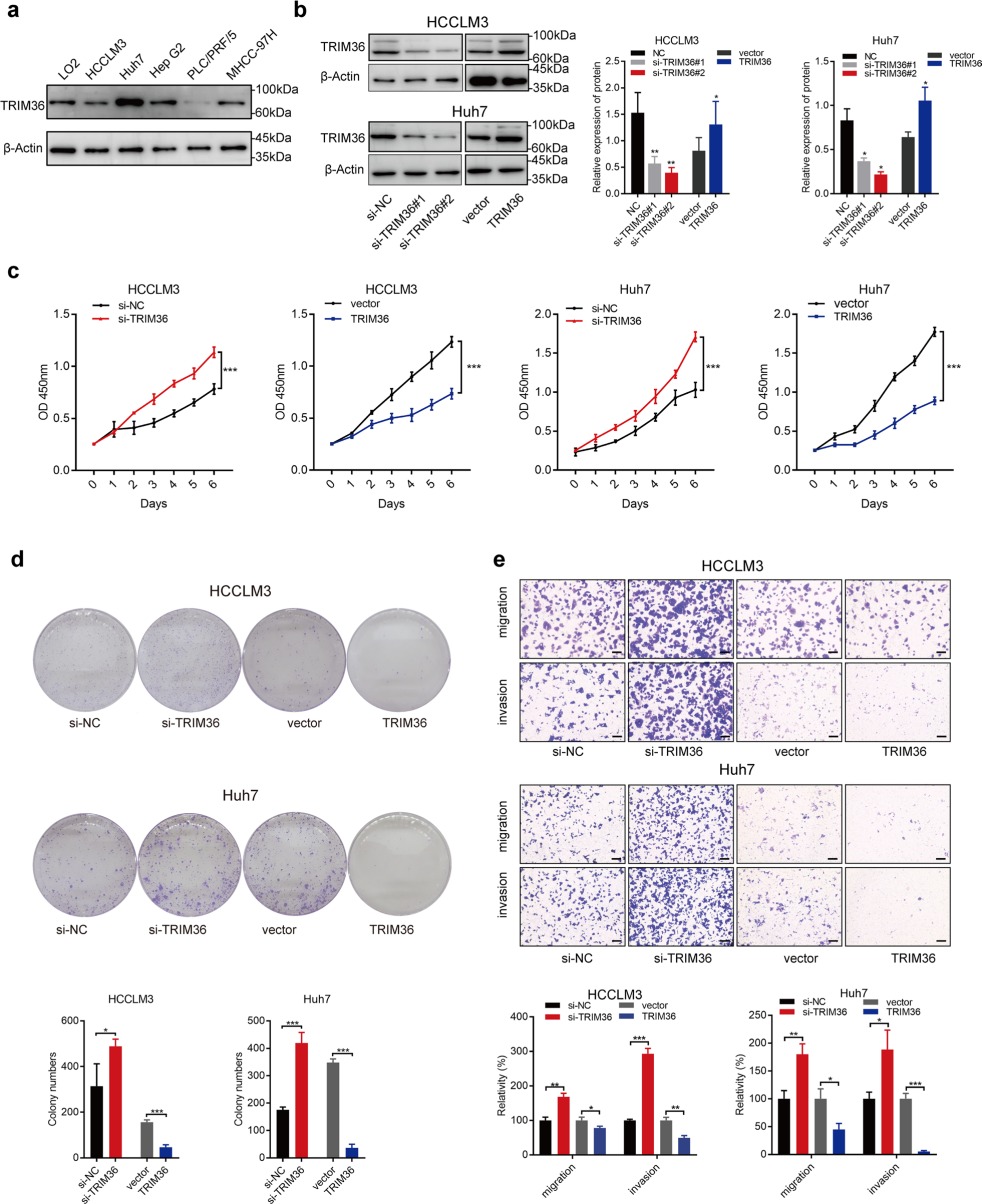
TRIM36 represses HCC cellular proliferation, migration, and invasion. **a** Western blotting analysis showing TRIM36 expression level in LO2, HCCLM3, Huh7, HepG2, PLC/PRF/5, and MHCC-97H cell lines. **b** Left panel of Western blotting analysis showing TRIM36 expression level in HCCLM3 and Huh7 transfected with si-NC, si-TRIM36#1 or si-TRIM36#2, and right panel of Western blotting analysis showing TRIM36 expression level in HCCLM3 and Huh7 transduced with vector or TRIM36. Bar graph showing TRIM36 expression in indicated groups. **c** Graph showing the proliferation ability measure by Cell counting kit-8 (CCK-8) in HCCLM3 and Huh7 cells transfected with si-NC or si-TRIM36 and HCCLM3 and Huh7 cells transduced with vector or TRIM36 at different timepoints (0 day, 1 day, 2 day, 3 day, 4 day, 5 day and 6 day). **d** colony formation images in HCCLM3 and Huh7 cells transfected with si-NC or si-TRIM36 and cells transduced with vector or TRIM36. Bar graph showing colony numbers in indicated groups. **e** Transwell migration and invasion assay showing the migration and invasion ability in HCCLM3 and Huh7 cells transfected with si-NC or si-TRIM36 and cells transduced with vector or TRIM36. Bar graph showing relative migration and invasion in indicated groups. Scale bars, 100 μm. *P* value was calculated using two-tailed unpaired Student’s *t*-test within two groups, and one-way ANOVA was used among three groups, error bars are means ± SD, n = 3 independent repeats. *P < 0.05, **P < 0.01, ***P < 0.001

The effect of TRIM36 regulation on tumor properties was assessed in both loss- and gain-of function experiments. Western blotting revealed the expression levels of TRIM36 in the normal control liver cell line LO2 and the liver cancer cell lines HCCLM3, Huh7, Hep G2, PLC/PRF/5, and MHCC-97H (Fig. 2a). TRIM36 expression was higher in Huh7 cells and relatively lower in HCCLM3 cells than in control LO2 cells. Therefore, Huh7 and HCCLM3 were selected for further experiments, in which si-TRIM36 was transfected into both lines, moreover, both lines were stably transduced with an adenovirus overexpressing TRIM36. Expression levels of TRIM36 in both lines were measured by western blotting and RT-qPCR (Fig. 2b and Additional file 4: Fig. S2a, b). Proliferation was measured using CCK-8 and colony formation assays. As shown in Fig. 2c and d, CCK-8 and colony formation assays identified that the proliferation of Huh7 and HCCLM3 cells increased with the downregulation of *TRIM36* compared to that in the control. In contrast, the proliferation of both lines overexpressing TRIM36 was lower than that of the vector control. Transwell invasion and migration assays (Fig. 2e) showed that the upregulation of TRIM36 significantly repressed both invasion and migration of Huh7 and HCCLM3 cells, while the downregulation of TRIM36 elevated both invasion and migration.

### TRIM36 promotes HCC apoptosis in a caspase-dependent manner

**Fig. 3:**
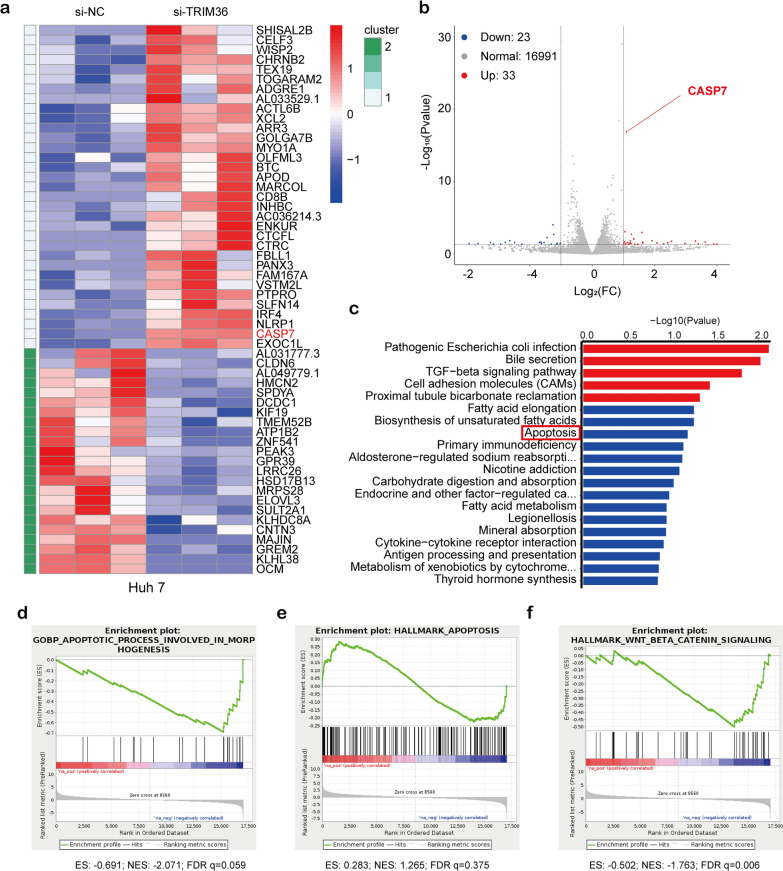
Bioinformation analysis of RNA-seq assays.** a** Heat map showing differentially expressed transcripts in Huh7 cells transfected with si-NC or si-TRIM36.** b** volcano plots showing differentially expressed transcripts in Huh7 cells transfected with si-NC or si-TRIM36.** c** GO analyses showing the enrichment of pathway. **d-f** GSEA showing enrichment of the apoptosis and WNT/β-catenin pathway controlled by TRIM36. ES = Enrichment score, NES = Normalized Enrichment Score

**Fig. 4:**
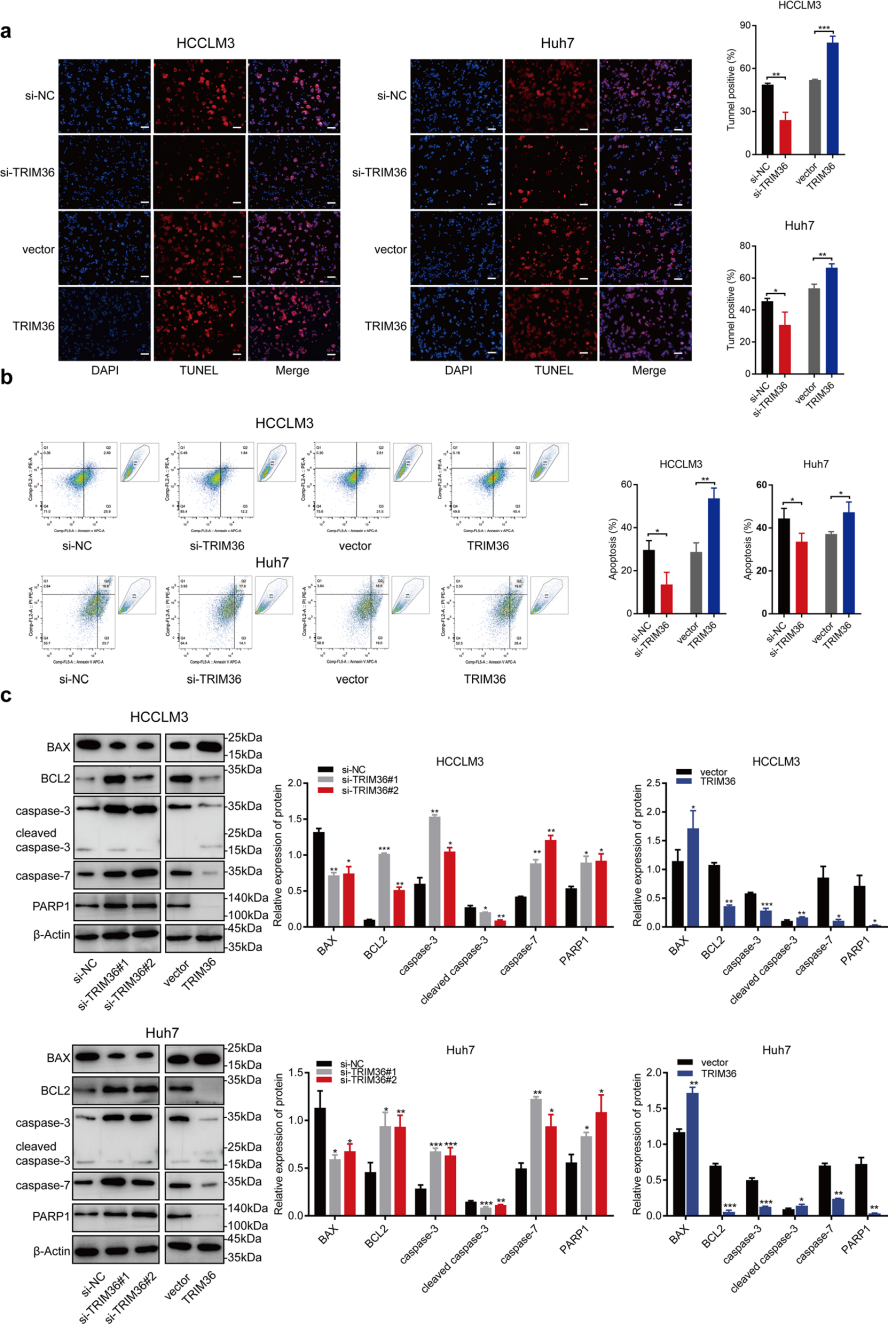
TRIM36 promotes HCC apoptosis in a caspase-dependent manner. **a** TUNEL assay images in HCCLM3 and Huh7 cells transfected with si-NC or si-TRIM36 and cells transduced with vector or TRIM36. The left blue panel representing nuclear stain, the median red panel representing TUNEL positive cells, and the right panel demonstrating the merge of the blue and the red picture. Scale bars, 100 μm. Bar graph showing the relative TUNEL positive cells in indicated groups. **b** Annexin V/PI staining assay showing the healthy cells, apoptotic cells, and dead cells in HCCLM3 and Huh7 cells transfected with si-NC or si-TRIM36 and cells transduced with vector or TRIM36. Bar graph showing the percentage of apoptosis cells in indicated groups. **c** Left panel of Western blotting analysis showing expression level of BAX, BCL-2, caspase-3, cleaved caspase-3, caspase-7, PARP1 in HCCLM3 and Huh7 transfected with si-NC, si-TRIM36#1 or si-TRIM36#2, and right panel of Western blotting analysis showing TRIM36 expression level in HCCLM3 and Huh7 transduced with vector or TRIM36. Bar graph showing relative expression of protein in indicated groups. *P* value was calculated using two-tailed unpaired Student’s *t*-test within two groups, and one-way ANOVA was used among three groups, error bars are means ± SD, n = 3 independent repeats. *P < 0.05, **P < 0.01, ***P < 0.001

**Fig. 5:**
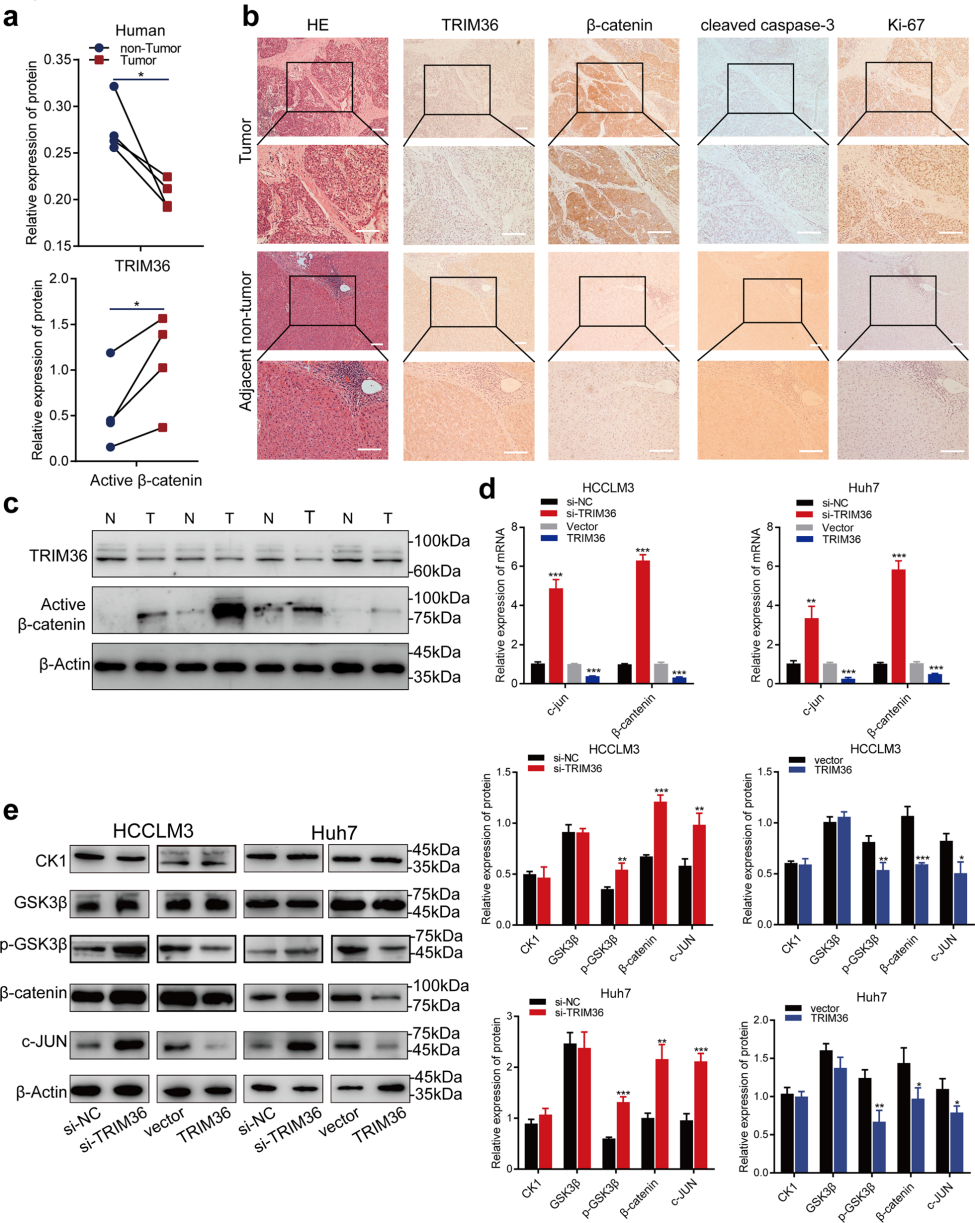
TRIM36 inhibits Wnt/β-catenin pathway. **a**, **c** Western blotting analysis showing TRIM36 and active β-catenin expression in HCC and adjacent non-tumor tissues, *p* value was calculated using two-tailed paired Student’s *t*-test, n = 4 independent repeats. **b** Left panel showing the hematoxylin–eosin staining of HCC and adjacent non-tumor tissues, the remaining set showing immunohistochemistry image of TRIM36, β-catenin, cleaved caspase-3, and Ki-67 in HCC and adjacent non-tumor tissues. Scale bars, 100 μm. The upper panel was 100 × magnification, the lower panel was 200 × magnification. **d** qRT–PCR showing the expression level of c-Jun and β-catenin mRNA in HCCLM3 and Huh7 transfected with si-NC, si-TRIM36, and cells transduced with vector or TRIM36. **e** Western blotting analysis showing expression level of CK1, GSK3β, p- GSK3β, c-Jun in HCCLM3 and Huh7 cells transfected with si-NC or si-TRIM36 and cells transduced with vector or TRIM36. Bar graph showing the relative protein expression in indicated groups. *P* value was calculated using two-tailed unpaired Student’s *t*-test, error bars are means ± SD, n = 3 independent repeats. *P < 0.05, **P < 0.01, ***P < 0.001

The versatile TRIM protein family exerts multiple cellular functions in multiple diseases. Thus, to further understand the core function of TRIM36 in HCC, RNA-Seq analysis was conducted using Huh7 cells with *TRIM36* knockdown and control cells transfected with si-NC. Heat maps and volcano plots showing differentially expressed transcripts are shown in Fig. 3a and b. Among these mRNAs, a statistically higher expression level of caspase-7 mRNA was found when *TRIM36* was knocked down in Huh7 cells. Furthermore, GO and KEGG analyses revealed a close association between apoptosis and TRIM36 (Fig. 3c). Consistent with GO and KEGG analyses, GSEA of the whole transcriptome showed enrichment of genes involved in apoptosis (Fig. 3d, e). As shown in Fig. 5d, IHC revealed decreased staining for cleaved caspase-3 in clinical HCC samples compared with that in pan-cancerous tissue. Further experiments were conducted in our study, firstly, TUNEL assays showed increased positive Huh7 and HCCLM3 cells overexpressing TRIM36 and a decreased number of TUNEL-positive cells in Huh7 and HCCLM3 cells with TRIM36 knockdown (Fig. 4a). Flow cytometry shown that in Huh7 and HCCLM3 cells overexpressing TRIM36, increased numbers of apoptotic cells were observed relative to controls. In contrast, a significantly lower extent of apoptosis was observed in Huh7 and HCCLM3 cells with TRIM36 knockdown (Fig. 4b). Immunoblotting (Fig. 4c) confirmed that caspase-3, caspase-7, and PARP1 were upregulated by transfection with si-TRIM36, while active (cleaved) caspase-3 were downregulated, compared to the control cells. We also observed downregulation of the pro-apoptotic protein BAX and upregulation of the anti-apoptotic protein BCL2 by transfection with si-TRIM36. In both cell lines stably overexpressing TRIM36, the converse result was observed, confirming the upregulation of apoptosis by TRIM36.

### TRIM36 regulates HCC proliferation, invasion, and migration through Wnt/β-catenin signaling

**Fig. 6:**
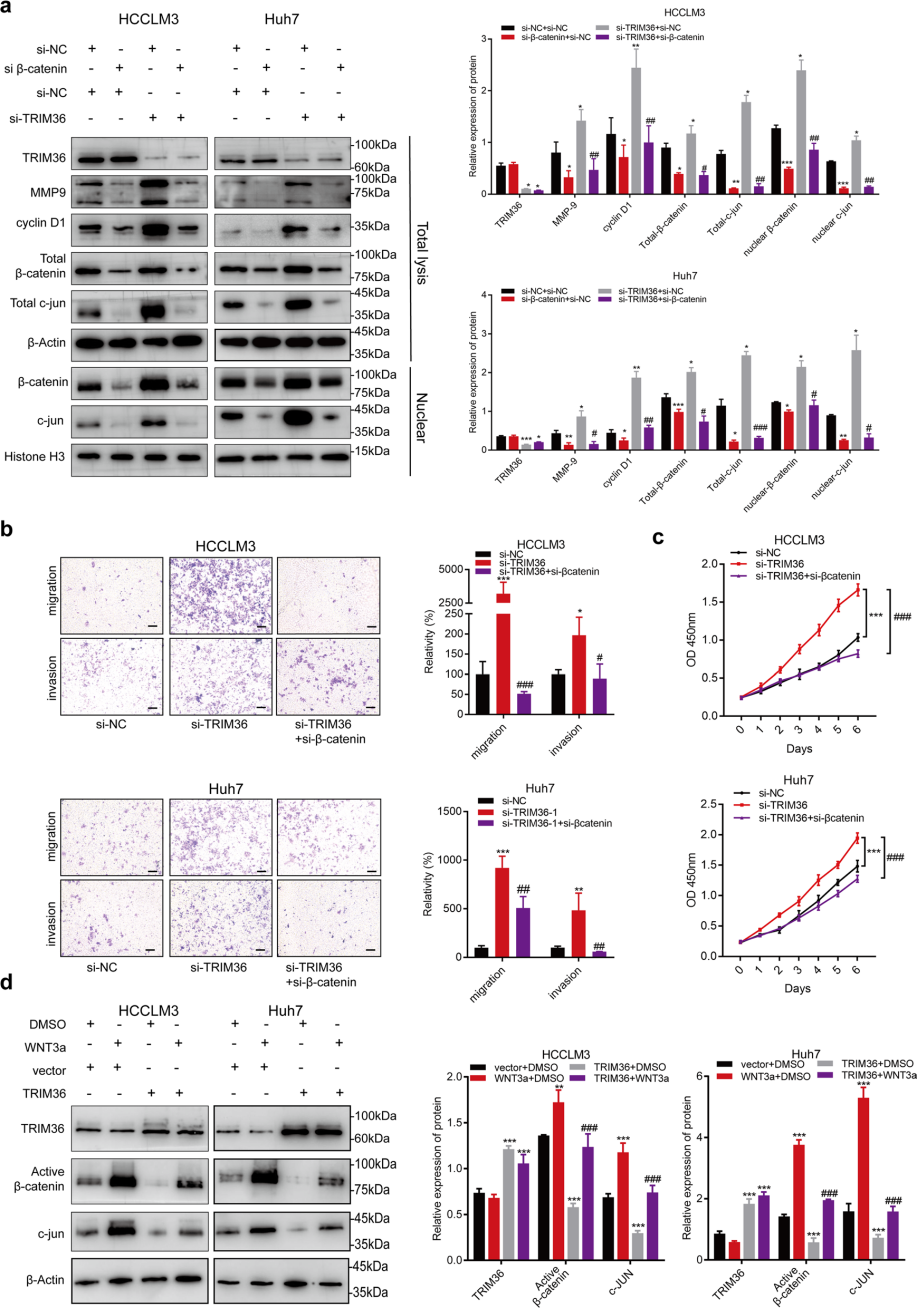
TRIM36 regulates cellular proliferation, invasion, and migration through the Wnt/β-catenin pathway. **a** Western blotting analysis showing expression level of TRIM36, MMP9, cyclinD1, total β-catenin, total c-Jun and expression level of nuclear c-jun and nuclear β-catenin in HCCLM3 and Huh7 cells transfected with si-NC, si β-catenin, si-TRIM36 and cells co-transfected with si-β-catenin and si-TRIM36. Bar graph showing the relative protein expression in indicated groups. **b** Transwell migration and invasion assay showing the migration and invasion ability in HCCLM3 and Huh7 cells transfected with si-NC, si-TRIM36 and co-transfected with si-β-catenin and si-TRIM36. Bar graph showing migration of invasion ability in indicated groups. Scale bars, 100 μm. **c** Graph showing the proliferation ability measure by Cell counting kit-8 (CCK-8) in HCCLM3 and Huh7 cells transfected with si-NC, si-TRIM36 and co-transfected with si-β-catenin and si-TRIM36 at different timepoints (0 day, 1 day, 2 day, 3 day, 4 day, 5 day and 6 day). **d** Western blotting analysis showing expression level of TRIM36, active β-catenin and c-Jun expression level in HCCLM3 and Huh7 cells treated with control, WNT3a (100 ng/mL), TRIM36 overexpression and cells co-treated with WNT3a (100 ng/mL) and TRIM36 overexpression. Bar graph showing the relative protein expression in indicated groups. *P* value was calculated using one-way ANOVA. *P < 0.05 vs NC group, **P < 0.01 vs NC group, ***P < 0.001 vs NC group; #*P* < 0.05 vs si-TRIM36 group or TRIM36, ##*P* < 0.01 vs si-TRIM36 group or TRIM36, ###*P* < 0.001 vs si-TRIM36 or TRIM36 group

GSEA data (Fig. 3f) confirmed the enrichment of the Wnt/β-catenin pathway by TRIM36. Consistently, immunoblotting and IHC analysis in clinical HCC tissue exhibited decreased expression for TRIM36 and increased expression for active β-catenin, β-catenin and Ki-67 compared to adjacent control tissue, further verifying the connection between TRIM36 and the Wnt/β-catenin pathway (Fig. 6a–c). Accordingly, we conducted a deeper examination of the correlation between the Wnt/β-catenin pathway and TRIM36. The results in Fig. 5 represented a elevated level of β-catenin and c-JUN in cells transfected with si-TRIM36; in cells transfected with overexpressed TRIM36 represented a decreased expression of β-catenin and c-JUN. The β-catenin destruction complex—consisting of axin, glycogen synthase kinase 3β (GSK3β), adenomatous polyposis coli protein (APC), and casein kinase I isoform-α (CK1α)—phosphorylates β-catenin for further proteasomal degradation, thus exerting an important regulatory function [33, 34]. As shown in Fig. 5**,** pGSK3β expression was increased in Huh7 and HCCLM3 cells treated with si-TRIM36, which might block the phosphorylation of β-catenin and contribute to accumulation and nuclear translocation of β-catenin [8, 33]; and pGSK3β expression was decreased in Huh7 and HCCLM3 cells with overexpressed TRIM36. Besides, the expression of GSK3β and CK1 shown no significant differences between groups. The results confirmed the inhibition of TRIM36 on WNT/β-catenin pathway, and implying the inhibition was conducted through regulation on β-catenin expression and activity of GSK3β.

Of notes, we conducted a rescue experiment in which Huh7 and HCCLM3 cells were transduced with control products, si-TRIM36, si-β-catenin, or a combination of si-TRIM36 and si-β-catenin. The overexpression of β-catenin, c-Jun, nuclear β-catenin, and nuclear c-JUN was validated in Huh7 and HCCLM3 cells transfected with si-TRIM36, compared to those in the control (Fig. 6a). MMP9 and cyclin-D1, two important downstream proteins of the Wnt/β-catenin pathway were upregulated in Huh7 and HCCLM3 cells transfected with si-TRIM36 (Fig. 6a). Functionally, compared with controls, Huh7 and HCCLM3 cells transfected with si-TRIM36 displayed elevated proliferation, invasion, and migration. Importantly, the activation of the Wnt/β-catenin pathway and cellular proliferation, invasion, and migration by si-TRIM36 were attenuated by cotransduction with si-β-catenin (Fig. 6b, c), which decreased β-catenin, c-Jun, MMP9 and cyclin-D1 expression. Cells co-transduced with si-β-catenin and si-TRIM36 displayed decreased proliferation with lower OD values and a decreased invasion and migration (Fig. 6b, c). In deeply, we further conducted immunoblotting assay in cells under the treatment of Wnt3a. HCCLM3 and Huh7 cells were treated with control products, TRIM36, WNT3a (100 ng/mL), or a combination of TRIM36 and WNT3a (100 ng/mL). The down-regulation of active β-catenin and c-Jun were detected in Huh7 and HCCLM3 cells transfected with TRIM36, compared to those in the control. Importantly, the inhibition of the Wnt/β-catenin pathway were reversed by WNT3a treatment (Fig. 6d). Data gathering together, we confirmed that TRIM36 represses proliferation, invasion, and migration by inhibiting the Wnt/β-catenin pathway.

### Inhibition of TRIM36 in vivo promotes tumor progression

**Fig. 7:**
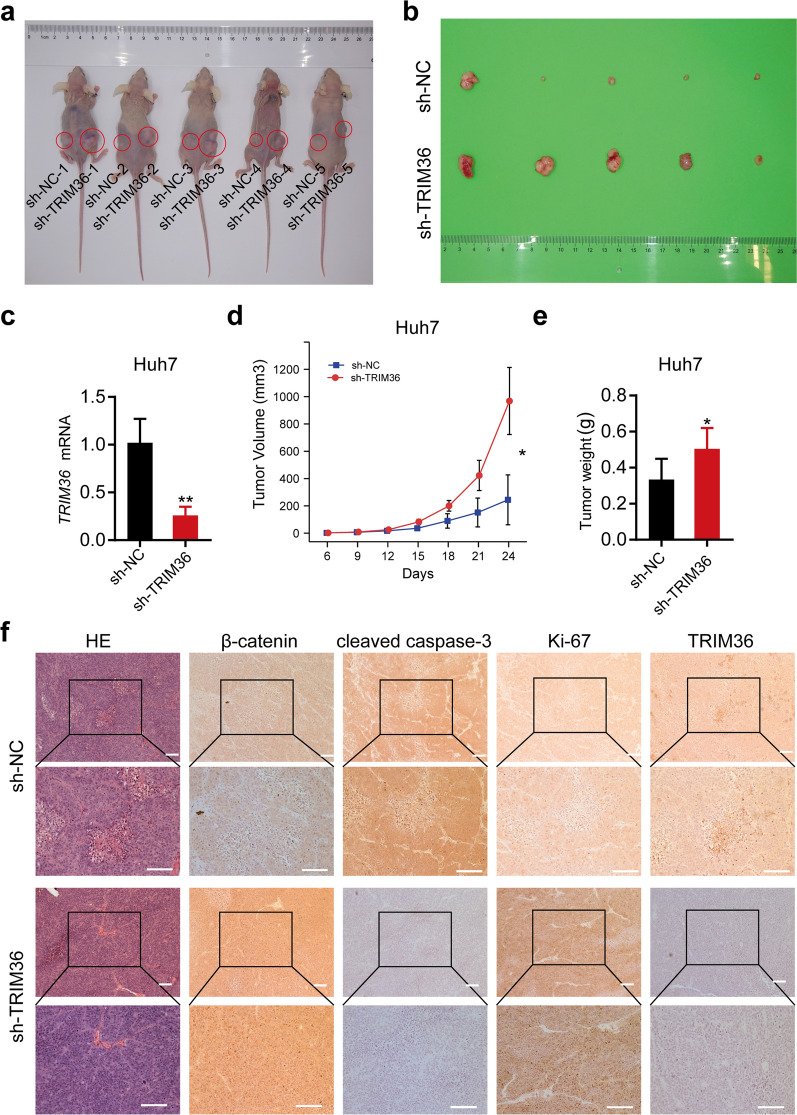
Inhibition of TRIM36 in vivo causes the progression of tumor growth.** a** and **b** Subcutaneous tumor formation in Huh7 xenograft tumors established via Huh7 cell line transduced with lenti-sh-NC and lenti-sh-TRIM36. **c** qRT–PCR showing the expression level of TRIM36 mRNA in Huh7 xenograft tumors established via Huh7 cell line transduced with lenti-sh-NC and lenti-sh-TRIM36. **d** Individual value plot showing the tumor volume of Huh7 xenograft tumors established via Huh7 cell line transduced with lenti-sh-NC and lenti-sh-TRIM36 at different time points (6 day, 9 day, 12 day, 15 day, 18 day, 21 day, 24 day). **e** Individual value plot showing the tumor weights of in Huh7 xenograft tumors established via Huh7 cell line transduced with lenti-sh-NC and lenti-sh-TRIM36. **f** Left panel showing the hematoxylin–eosin staining of tumor mass established via Huh7 cell line transduced with lenti-sh-NC and lenti-sh-TRIM36, and the remaining showing immunohistochemical staining of β-catenin, cleaved caspase-3, Ki-67 and TRIM36 in HCC and adjacent non-tumor tissues. Scale bars, 100 μm. The upper panel was 100 × magnification, the lower panel was 200 × magnification. *P* value was calculated using two-tailed unpaired Student’s *t*-test, error bars are means ± SD, n = 5 independent repeats. *P < 0.05, **P < 0.01

To further confirm the repressive role of TRIM36 in HCC progression, we constructed a stable *TRIM36* knockdown Huh7 cell line. Cells transfected with lenti-sh-TRIM36 or vector were xenografted (Fig. 7a, b). Tumor tissues were collected for IHC, which confirmed the decreased expression of TRIM36 (Fig. 7f). As shown in Fig. 7c–e, cells expressing sh-TRIM36 produced significantly larger lesion sizes, tumor volumes, and tumor weights compared to the vector-only control. IHC also revealed an elevated level of β-catenin and Ki-67 staining in the sh-TRIM36-expressing cells compared with controls. Decreased cleaved caspase-3 staining was also observed in sh-TRIM36-expressing cells (Fig. 7f). Data gathering together further confirmed the regulation of TRIM36 on WNT/β-catenin pathway and apoptosis identified in vitro.

## Discussion

**Fig. 8:**
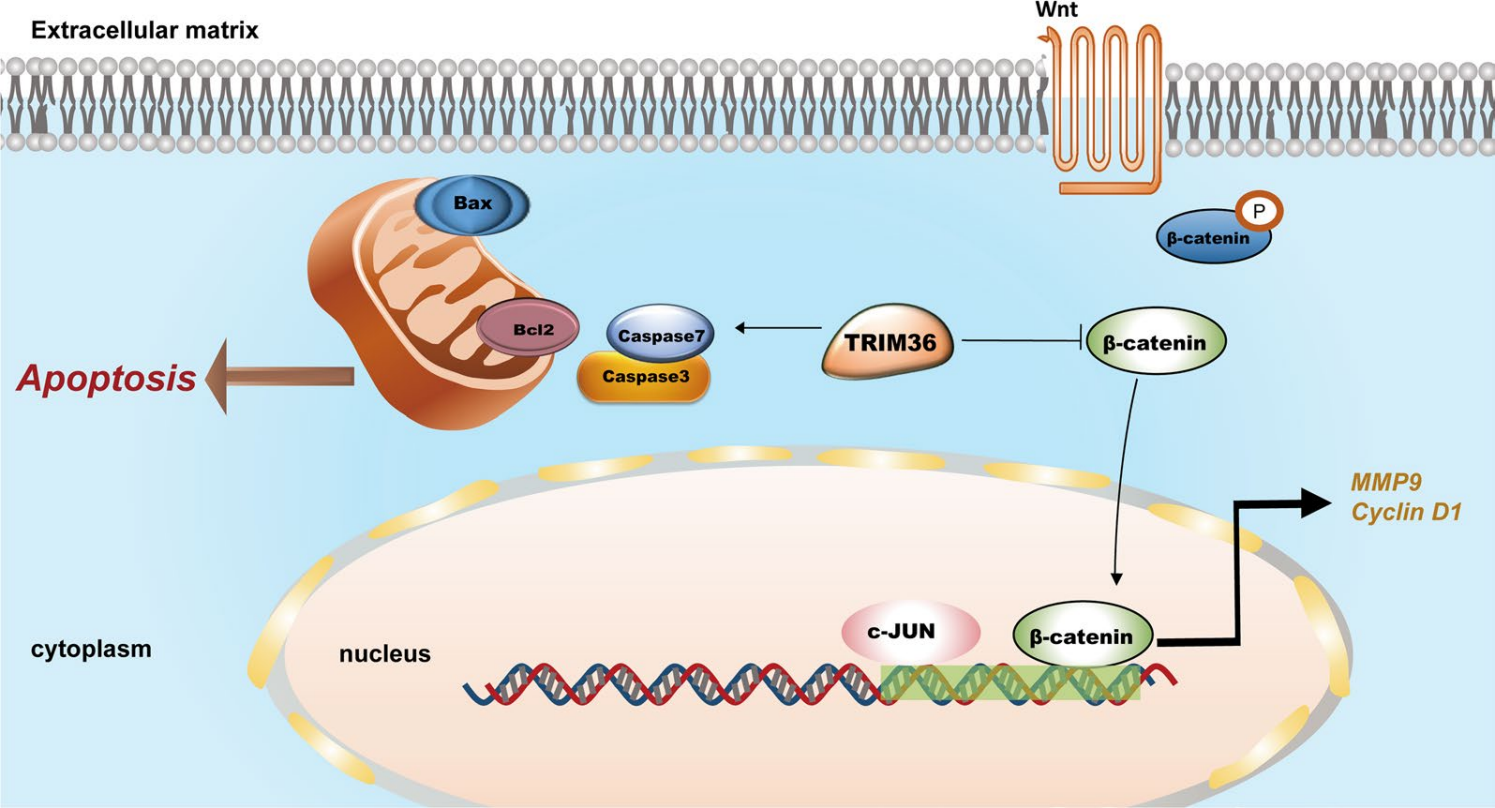
The working model describes the mechanism of TRIM36 in HCC, which shows that TRIM36 upregulates apoptosis and inhibits tumorigenesis via the WNT/β-catenin pathway

Despite progressive advances in the treatment of HCC, with advanced diagnosis and poor prognosis, HCC remains the leading cause of cancer-related deaths worldwide [5, 13]. A profound understanding of tumorigenesis and accelerated validation of promising therapeutic targets and novel biomarkers are yet to be developed. Our study is the first to indicate that TRIM36 acts as a tumor suppressor in HCC. In our study, TRIM36 expression was significantly downregulated in HCC tissues and correlated with poor clinical outcomes in HCC. TRIM36 was identified to play a stimulatory role in caspase-dependent apoptosis in our study. And TRIM36 significantly decreased proliferation, invasion, and migration via Wnt/β-catenin pathway. Here, we provide a comprehensive and detailed molecular portrait of the regulated pathway and mechanism underlying the tumor-suppressive effects of TRIM36 in HCC (Fig. 8).

Apoptosis is an elaborate caspase-dependent process of programmed cell death; its dysfunction contributes to tumor initiation [35, 36]. Caspase-3 and caspase-7 are effectors in the apoptosis cascade, providing feedback amplification of apoptosis signals [37]. In our study, increased expression of caspase-7 and genes involved in apoptosis was shown using RNA-seq and bio-functional analysis in Huh7 cells with *TRIM36* knockdown, apoptosis assays highlighted the role of TRIM36 in apoptosis. More significantly, consistent with our data, compatible regulation of apoptosis-related pathways has been observed in PC cells overexpressing TRIM36 [27]. Previous research has also revealed that some homologous regulation of apoptosis is performed by proteins in the TRIM family; for example, hepatocyte TRIM27 deficiency has been shown to contribute to resistance to apoptosis [38]. *TRIM8* knockdown downregulated cleaved caspase-3 expression [39], and TRIM34-mediated apoptosis in HEK293T cells [40].

Reactivation of Wnt–β-catenin signaling promotes the transcription of genes involved in proliferation, migration, and metastasis in HCC [41–43] Moreover, chemoresistance, radiotherapy resistance, and tumor microenvironment are influenced by Wnt/β-catenin [44, 45]. As indicated in our study, regulation of Wnt/β-catenin signaling was further confirmed in Huh7 and HCCLM3 cells with TRIM36 knockdown or overexpression. Functionally, we confirmed that si-TRIM36 boosted the Wnt/β-catenin pathway, resulting in enhanced proliferation, migration, and invasion abilities of HCCLM3 and Huh7 cell lines. Similarly, other TRIM proteins, such as TRIM37, have also been found to inhibit the proliferation and invasion of platelet-derived growth factor BB-stimulated airway smooth muscle cells through the Wnt/β-catenin signaling [46]. In vivo, sh-TRIM36 treatment significantly inhibited HCC xenograft tumor growth, suggested that TRIM36 functions as a tumor suppressor. In addition, consistent with the in vitro results, H&E staining also revealed the upregulated expression of β-catenin and Ki-67, as well as downregulated cleaved caspase-3 in the sh-TRIM36 group compared with those in the control group. Furthermore, we found that TRIM36 upregulates apoptosis and represses the Wnt/β-catenin pathway in HCC.

Our study has several limitations. We used a tumor-derived cell line xenograft model, which is widely used and has been verified to retain most of the features of primary HCC, to mimic HCC and further verify our findings in vivo. However, this model fails to mimic the convoluted tumor microenvironment [38]. Although substantial resources have been allocated to study HCC, extensive obstacles remain, and further studies are needed to gain a deeper understanding of the underlying relationship between TRIM proteins and HCC.

## Conclusions

Consistently, TRIM36 expression is downregulated; this decrease is correlated with poor clinical outcomes in HCC. TRIM36 activates apoptosis and inhibits cellular proliferation, invasion, and migration via the Wnt/β-catenin pathway. These data collectively suggest that TRIM36 functions as a tumor suppressor, which may serve as an important biomarker and promising therapeutic target for HCC.

## Supplementary Information

**Additional file 1.**

**Additional file 2.**

**Additional file 3: Figure S1.** Cox analysis of recurrence-free survival (RFS) **a, b** Univariate and multivariate Cox analysis of RFS

**Additional file 4: Figure S2.** a, b qRT-PCR showing the expression level of TRIM36 mRNA in HCCLM3 and Huh7 transfected with si-NC, si-TRIM36#1 or si-TRIM36#2, and cells transduced with vector or TRIM36. *P* value was calculated using two-tailed unpaired Student’s *t*-test within two groups, and one-way ANOVA was used among three groups, error bars are means ± SD, n = 3 independent repeats. **P < 0.01, ***P < 0.001.

## Data Availability

All data generated or analyzed in this study are included in this published article and its additional files.
