# Supplementary material for: TRIM36 inhibits tumorigenesis through the Wnt/β-catenin pathway and promotes caspase-dependent apoptosis in hepatocellular carcinoma
Source: Cancer Cell Int. 2022 Sep 6;22:278. doi: 10.1186/s12935-022-02692-x (PMC9450375; doi:10.1186/s12935-022-02692-x)
Supplement: Supplementary file 1 — Additional file 1. [file 12935_2022_2692_MOESM1_ESM.docx]

Supplementary Table 1

| Oligonucleotides | |
| --- | --- |
| qRT-PCR: TRIM36 | F AACATGTCGGAGTCTGGGGA |
|  | R GTGGCAAGTTCCGTCGTCTT |
| qRT-PCR: c-JUN | F CCTGCGTCTTAGGCTTCTCC |
|  | R CCTGCGTCTTAGGCTTCTCC |
| qRT-PCR: β-catenin | F GGCCCAGAATGCAGTTCGCCTT |
|  | R AATGGCACCCTGCTCACGCA |
| qRT-PCR: β-Actin | F CTCGCTGTCCACCTTCCA |
|  | R ACCTTCACCGTTCCAGTTTT |
| si-TRIM36-1 | TGAGTGAATTTGGCTACAT |
| si-TRIM36-2 | CATGGAATTGATAGCTAAA |
| si-β-catenin | sense 5’GAUGGUGUCUGCUAUUGUA dTdT 3’  antisense 3’dTdT CUACCACAGACGAUAACAU 5’ |
