# Supplementary material for: TRIM36 inhibits tumorigenesis through the Wnt/β-catenin pathway and promotes caspase-dependent apoptosis in hepatocellular carcinoma
Source: Cancer Cell Int. 2022 Sep 6;22:278. doi: 10.1186/s12935-022-02692-x (PMC9450375; doi:10.1186/s12935-022-02692-x)
Supplement: Supplementary file 2 — Additional file 2. [file 12935_2022_2692_MOESM2_ESM.docx]

| Software and Algorithms | | |
| --- | --- | --- |
| Graphpad Prsim 8 | GraphPad | www.graphpad.com/scientific-software/prism/ |
| ImageJ software | ImageJ open source | http: imagej.net/Welcome |
| FlowJo | FlowJo LLC | www.flowjo.com |
| SPSS26.0 | IBM SPSS Statistics | www.spss.com |
| GSEA |  | http:software.broadinstitute.org/gsea/index.jsp |

Supplementary Table 2
