# Supplementary figures and images for: TRIM36 inhibits tumorigenesis through the Wnt/β-catenin pathway and promotes caspase-dependent apoptosis in hepatocellular carcinoma
Source: Cancer Cell Int. 2022 Sep 6;22:278. doi: 10.1186/s12935-022-02692-x (PMC9450375; doi:10.1186/s12935-022-02692-x)

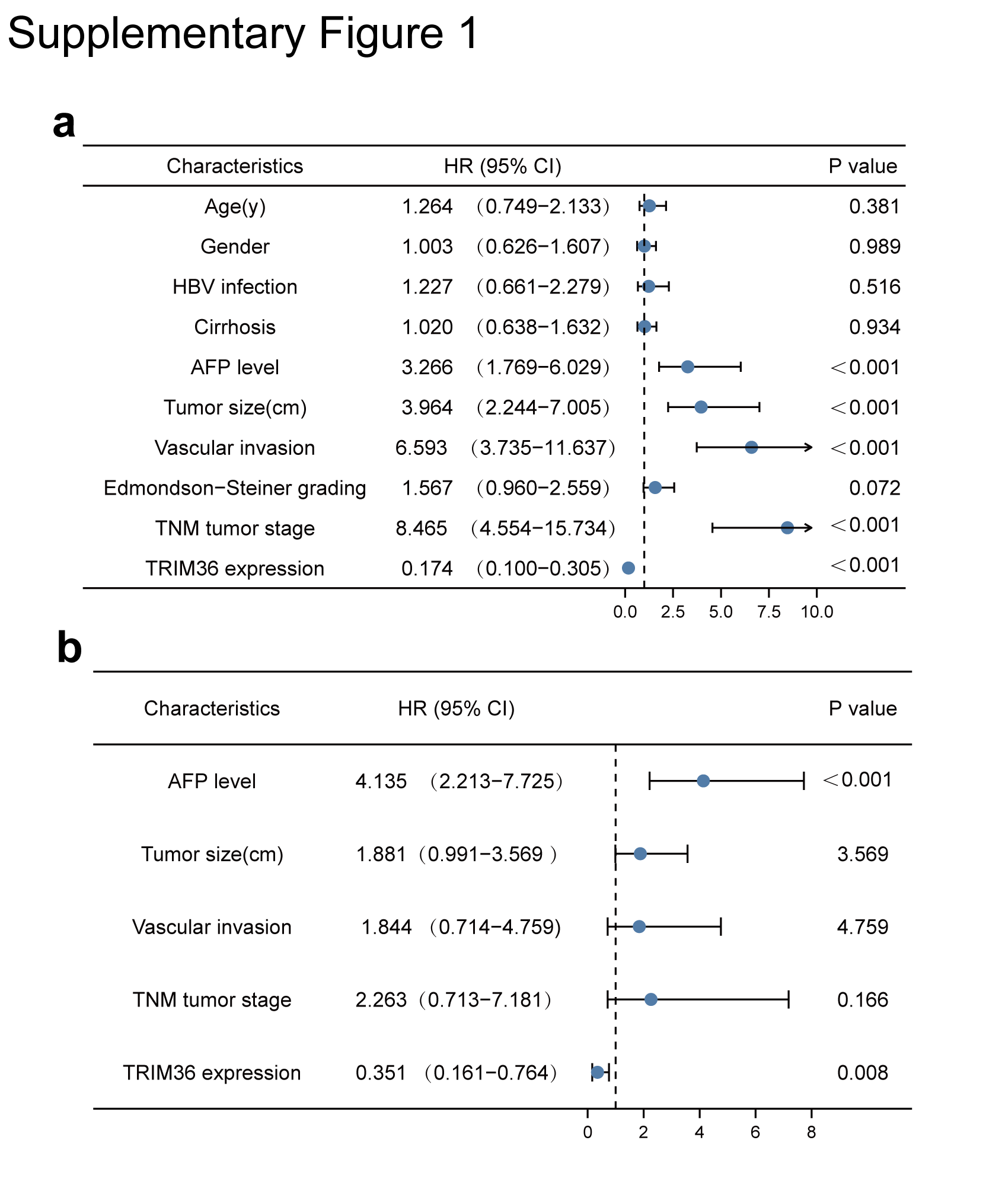

Supplement: Supplementary file 3 — Additional file 3: Figure S1. Cox analysis of recurrence-free survival (RFS) a, b Univariate and multivariate Cox analysis of RFS [file 12935_2022_2692_MOESM3_ESM.tif]

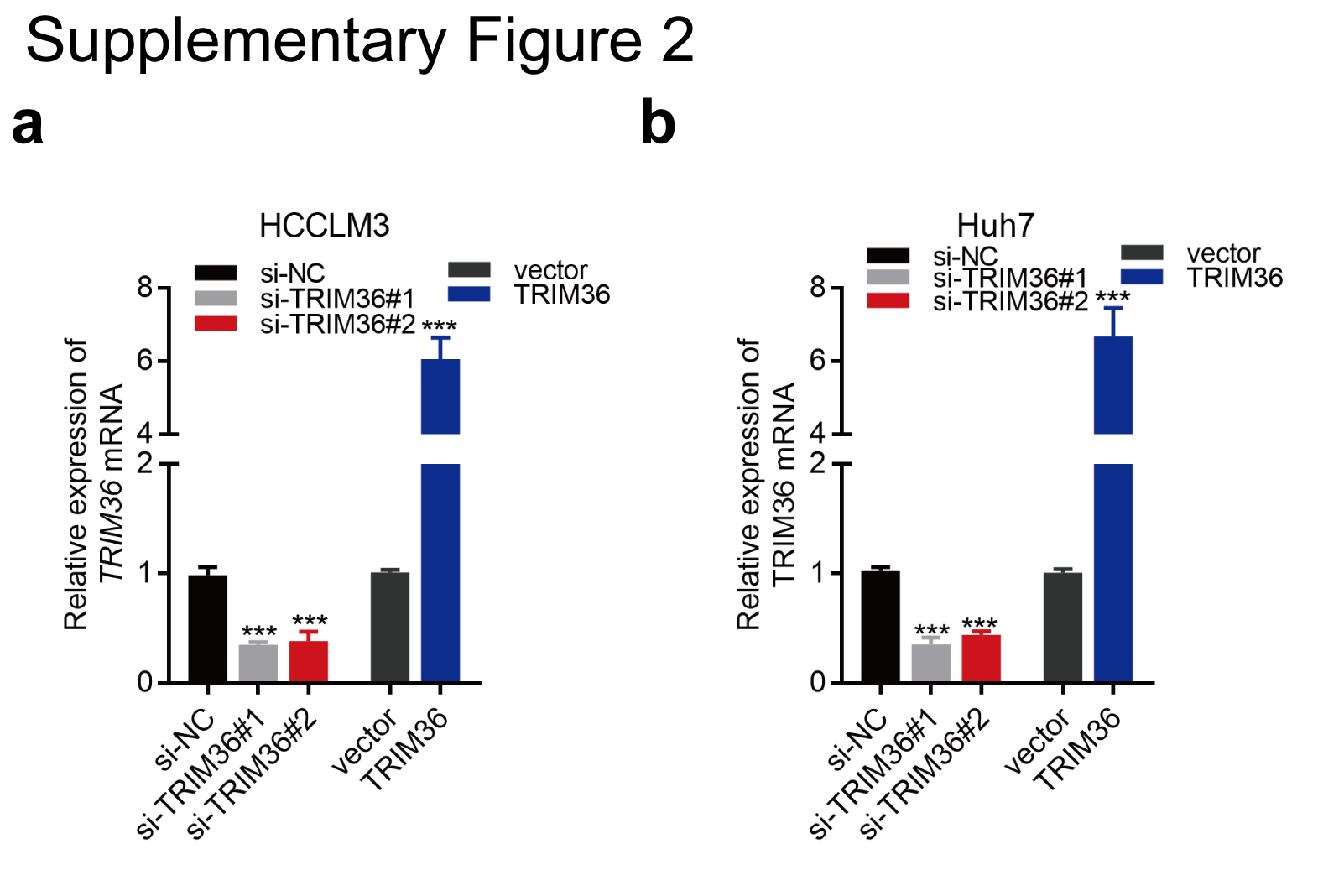

Supplement: Supplementary file 4 — Additional file 4: Figure S2. a, b qRT-PCR showing the expression level of TRIM36 mRNA in HCCLM3 and Huh7 transfected with si-NC, si-TRIM36#1 or si-TRIM36#2, and cells transduced with vector or TRIM36. P value was calculated using two-tailed unpaired Student’s t-test within two groups, and one-way ANOVA was used among three groups, error bars are means ± SD, n = 3 independent repeats. **P < 0.01, ***P < 0.001. [file 12935_2022_2692_MOESM4_ESM.tif]
